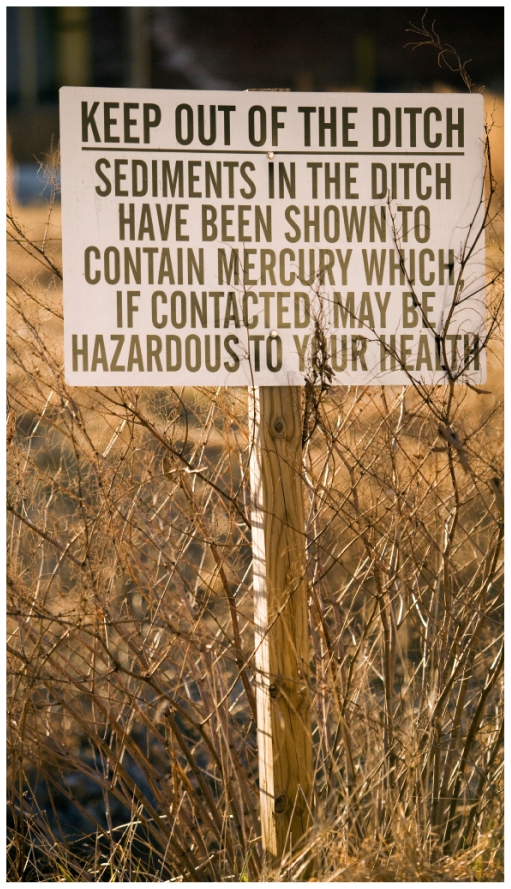# AIR POLLUTION: Mercury Emissions Not Shrinking as Forecast

**DOI:** 10.1289/ehp.118-a198

**Published:** 2010-05

**Authors:** David C. Holzman

**Affiliations:** **David C. Holzman** writes on science, medicine, energy, economics, and cars from Lexington and Wellfleet, MA. His work has appeared in *Smithsonian*, *The Atlantic Monthly*, and the *Journal of the National Cancer Institute*

Although amendments to the Clean Air Act in 1990 gave the U.S. Environmental Protection Agency (EPA) the authority to regulate mercury in coal-fired power plant emissions, no such rules are currently in place. Mercury emissions from coal-fired power plants nevertheless declined somewhat in the years directly after the act was amended. But in the March 2010 report *Dirty Kilowatts: America’s Top Fifty Power Plant Mercury Polluters*, the nonprofit Environmental Integrity Project (EIP) shows emissions have remained fairly steady since 2000, hovering between roughly 44 and 48 tons per year, despite the existence of technology that could drastically reduce the amount of mercury emitted from smokestacks.

The EPA has estimated that coal-fired power plants are the source of about 40% of anthropogenic mercury emissions in the United States. Worldwide they account for an estimated 25% of anthropogenic mercury emissions, according to *The Global Atmospheric Mercury Assessment: Sources, Emissions and Transport*, a 2008 report by the United Nations Environment Programme (UNEP). The UNEP authors note, “Mercury control technology for coal-fired power plants capable of capturing up to 95% of the mercury has only recently become commercially available and very few governments require it. Thus currently it is found on only a handful of plants [worldwide].”

In *Dirty Kilowatts*, the EIP reports that between 2007 and 2008, total emissions from the top 50 mercury-releasing power plants in the United States—of some 470 with sufficient emissions to be followed in the EPA’s Toxics Release Inventory (TRI)—fell by 0.26%, while total mercury emissions from all 470 plants fell by 4.71%. At many individual plants, however, mercury emissions actually rose, in one case by more than 100%. TRI data are self-reported by the utilities, who estimate emissions based on the amount of coal burned, the effectiveness of control devices, and characteristics of the coal, among other factors.

Some cases of increased emissions were probably due to switching coal sources, according to the report—burning bituminous coal like that mined in Appalachia typically releases less mercury than, for example, burning the same amount of sub-bituminous coal like that mined in Wyoming. But increased electricity demand also contributed to increased emissions, says Leonard Levin, technical executive of the Electric Power Research Institute in Palo Alto. In most cases, emissions increased or decreased with the quantity of coal burned, he says.

Elemental mercury emitted from power plants can reside in the atmosphere for 6 months to 2 years, and can travel from one hemisphere to another before being deposited on Earth’s surface, says Susan Keane, a senior environmental analyst at the Natural Resources Defense Council. For the general public, both U.S. and worldwide, the biggest source of mercury exposure is eating contaminated fish, says Keane. And while fetuses and young children are most sensitive to mercury poisoning, in her 2009 book, *Diagnosis: Mercury—Money, Politics & Poison*, San Francisco internist Jane M. Hightower recounts discovering mercury poisoning in adult patients who complained of headaches, cognitive difficulties such as trouble concentrating, stomach upsets, hair loss, and other symptoms.

The EIP report notes the 4.71% decline in mercury emissions is “nowhere near the levels that would be achieved if all plants installed modern pollution controls.” In 2005, when the EPA adopted a mercury cap-and-trade scheme in the form of the Clean Air Mercury Rule, the agency predicted mercury emissions from coal-fired power plants would fall to 31–34 tons by 2010 and could further drop to 15 tons with the use of “maximum achievable control technology” (MACT)—control devices, best work practices, and other methods that reduce emissions as much as possible.

In 2008 the U.S. Court of Appeals for the DC Circuit overturned the Clean Air Mercury Rule because the reductions sought would not have been achieved until 2018, says EIP attorney Ilan Levin. But new regulatory action is under way; the EPA intends to propose air toxics standards for coal-fired power plants by March 2011 and finalize a rule by the following November. Existing plants will need to reduce mercury emissions to levels now attained by the best-performing 12% of all similar sources, and new plants must incorporate MACT. One of the highest mercury emitters listed in the EIP report, Luminant, has already installed sorbent injection systems—state of the art for mercury mitigation—on 10 of its 12 coal-fired plants, says a company spokeswoman.

Meanwhile, UNEP has been working to negotiate global mercury emissions reductions among 140 participant nations. The United States had long resisted these efforts, preferring a voluntary approach to reducing mercury emissions. So when the U.S. government delegation announced they were ready to negotiate a treaty at the February 2009 biannual meeting of UNEP’s Governing Council, Keane says, “You’ve never seen a thousand jaws drop to the ground faster.” Following the United States’ lead, other recalcitrant countries came onboard to negotiate a legally binding instrument to control mercury pollution by the end of 2013, she says.

## Figures and Tables

**Figure:**